# Supplementary material for: An Improved Penalty-Based Excited-State Variational Monte Carlo Approach with Deep-Learning Ansatzes
Source: J Chem Theory Comput. 2024 Aug 30;20(18):7922–35. doi: 10.1021/acs.jctc.4c00678 (PMC11428158; doi:10.1021/acs.jctc.4c00678)
Supplement: Supplementary file 1 — ct4c00678_si_001.pdf [file ct4c00678_si_001.pdf]

Supporting Information: An Improved Penalty-Based Excited State Variational Monte Carlo Approach with Deep-Learning Ansatzes

P. Bernát Szabó,[†,‖] Zeno Schätzle,[†,‖] Michael T. Entwistle,[†] and Frank Noé[*,†,‡,¶,§]

†*Department of Mathematics and Computer Science, FU Berlin, Arnimallee 6, Berlin 14195, Germany*

‡*Microsoft Research AI4Science, Karl-Liebknecht Str. 32, 10178 Berlin, Germany*

¶*Department of Physics, FU Berlin, Arnimallee 14, Berlin 14195, Germany*

§*Department of Chemistry, Rice University, Houston, Texas 77005, United States*

‖*Equal contribution.*

E-mail: frank.noe@fu-berlin.de

S1 Numerical results

S1.1 Atoms

Table S1: Total energies in atomic units predicted by the improved penalty-based VMC method using the Psiformer ansatz for the atomic systems.

Atom	Total energy	Atom	Total energy	Atom	Total energy
Li	-7.47798(2)	N	-54.5888(3)	Ge	-3.74449(1)
	-7.41002(4)		-54.4993(1)		-3.74449(2)
	-7.41000(3)		-54.4990(1)		-3.74444(2)
	-7.40993(3)		-54.4984(1)		-3.71640(2)
	-7.3538(1)		-54.4981(1)		-3.71637(2)
	-7.3365(1)		-54.4980(5)		-3.71637(2)
	-7.33495(6)		-54.454(1)		-3.71632(2)
	-7.33482(5)		-54.4531(1)		-3.71632(2)
	-7.33399(6)		-54.4523(2)		-3.67453(2)
	-7.33283(5)		-54.2085(1)		-3.57472(1)
Be	-14.66730(3)	O	-75.06609(8)	As	-6.16580(1)
	-14.56719(2)		-75.0655(3)		-6.11444(2)
	-14.56714(3)		-75.0653(1)		-6.11442(2)
	-14.56713(2)		-74.997(6)		-6.11442(2)
	-14.47345(4)		-74.9928(3)		-6.11436(2)
	-14.47329(4)		-74.9925(5)		-6.11431(3)
	-14.47329(4)		-74.9925(2)		-6.08056(4)
	-14.42993(3)		-74.9919(8)		-6.08056(3)
	-14.4169(1)		-74.9108(8)		-6.08052(4)
	-14.40798(5)		-74.73027(7)		-5.92674(2)
B	-24.65280(9)	F	-99.732(1)	Se	-9.30018(2)
	-24.6528(1)		-99.7307(8)		-9.30018(2)
	-24.653(1)		-99.7303(3)		-9.30014(2)
	-24.52194(4)		-99.2651(3)		-9.26053(3)
	-24.52189(5)		-99.2641(2)		-9.26047(3)
	-24.52180(3)		-99.264(1)		-9.26044(3)
	-24.4707(4)		-99.2530(6)		-9.26039(3)
	-24.4341(1)		-99.253(1)		-9.26023(3)
	-24.4336(1)		-99.2527(7)		-9.20178(3)
	-24.4332(1)		-99.205(6)		-9.08504(2)
C	-37.8447(1)	Ne	-128.9344(4)		
	-37.8447(2)		-128.320(2)		
	-37.84436(6)		-128.3186(3)		
	-37.7982(1)		-128.3179(5)		
	-37.79785(9)		-128.3169(4)		
	-37.7976(4)		-128.3166(3)		
	-37.7975(1)		-128.3157(9)		
	-37.79734(9)		-128.250(2)		
	-37.7452(9)		-128.2478(6)		
	-37.69165(8)		-128.248(2)		

S1.2 Molecules

Table S2: Total energies in atomic units, and oscillator strengths predicted by the improved penalty-based VMC method using the Psiformer ansatz for the molecular systems.

	Total energy	Osc. str.		Total energy	Osc. str.		Total energy	Osc. str.
	-7.87794(1)	—		-113.3220(2)	—		-15.59737(3)	—
	-7.75952(2)	0.0000		-113.0912(2)	0.0000		-15.32636(3)	0.0000
LiH	-7.7468(1)	0.0052	CO	-113.0909(2)	0.0000	HCl	-15.32631(3)	0.0000
	-7.72519(2)	0.0000		-113.0103(7)	0.0898		-15.30740(3)	0.0219
	-7.72517(2)	0.0000		-113.0091(4)	0.0880		-15.30722(4)	0.0218
	-14.91745(3)	—		-78.5847(2)	—		-11.38910(3)	—
	-14.82519(3)	0.0629		-78.4182(2)	0.0000		-11.17752(3)	0.0000
BeH	-14.82516(4)	0.0630	C_2H_4	-78.3160(1)	0.0000	H_2S	-11.17134(3)	0.0000
	-14.7040(2)	0.0408		-78.3103(3)	0.0776		-11.16382(3)	0.0000
	-14.7024(1)	0.0372		-78.298(5)	0.3567		-11.15732(3)	0.0596
	-25.28907(3)	—		-114.505(1)	—		-43.1104(2)	—
	-25.2405(1)	0.0000		-114.3723(3)	0.0000		-43.0385(1)	0.0000
BH	-25.24041(4)	0.0000	CH_2O	-114.3567(6)	0.0000	H_2CSi	-43.0327(1)	0.0001
	-25.18363(4)	0.0236		-114.2409(9)	0.0000		-43.0186(3)	0.0000
	-25.18358(4)	0.0235		-114.2352(4)	0.0207		-43.0025(1)	0.0000
	-76.438(1)	—		-130.4747(2)	—		-49.45845(8)	—
	76.1680(6)	0.0000		-130.4460(7)	0.0000		-49.3883(1)	0.0000
H_2O	76.1531(2)	0.0505	HNO	-130.4083(4)	0.0046	CH_2S	-49.379(2)	0.0001
	76.0944(3)	0.0000		-130.3142(3)	0.0027		-49.33399(8)	0.0000
	76.0878(4)	0.0001		-130.2538(4)	0.0000		-49.24628(9)	0.0000
	-124.6746(4)	—		-138.411(2)	—		-232.2330(4)	—
	-124.5411(6)	0.0000		-138.3784(3)	0.0000		-232.0765(7)	0.0000
BF	-124.5408(3)	0.0000	HCF	-138.3166(4)	0.0125	C_6H_6	-232.0495(9)	0.0000
	-124.4410(6)	0.2948		-138.1694(4)	0.0000		-232.0479(9)	0.0000
	-124.4371(3)	0.3766		-138.153(1)	0.0547		-232.042(1)	0.0004

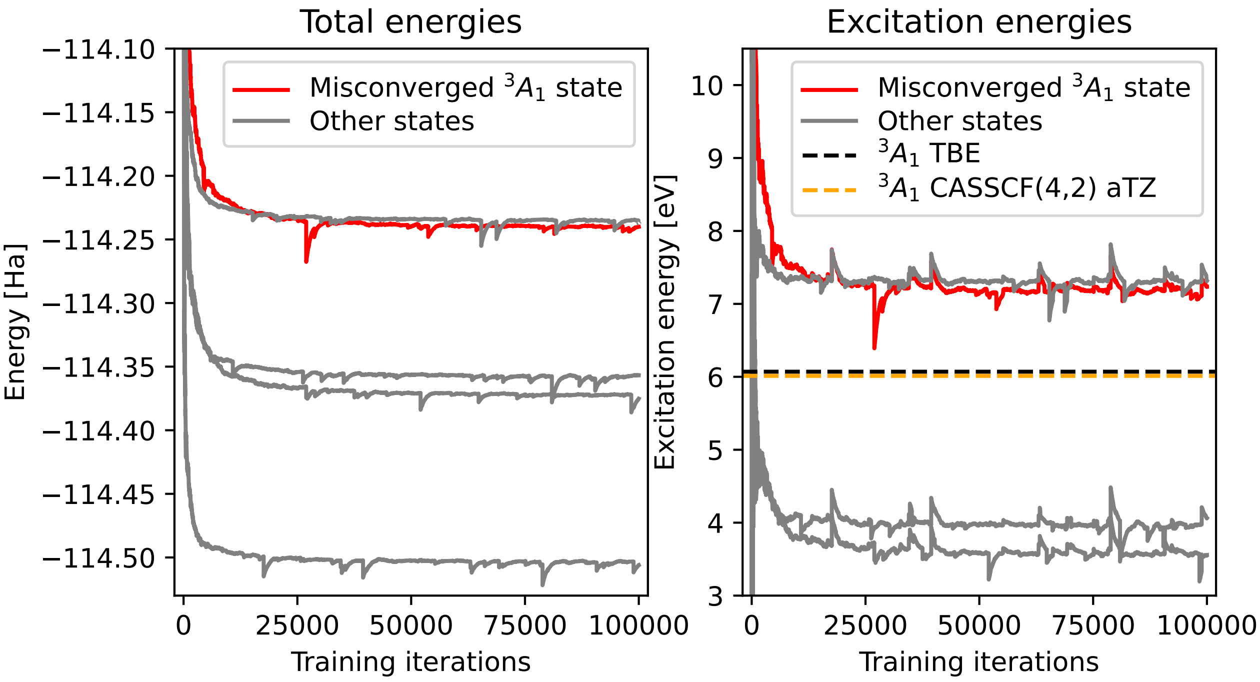

Figure S1: Convergence of the total and excitation energies of formaldehyde. Gray lines denote the correctly converged states, while red shows the state that should converge to $^3A_1$ but instead converges to the higher lying $^3B_2$ state. The theoretical best for the $^3A_1$ excitation energy is shown with black dashed line, while the excitation energy predicted by the baseline CASSCF solution is shown in orange.

S1.3 Carbon dimer

Table S3: Total energies in atomic units predicted by the improved penalty-based VMC method using the Psiformer ansatz for singlet excited states along the carbon dimer dissociation curve. Bond lengths are given in Ångström

Bond length	$X^1\Sigma_g^+$	$A^1\Pi_u$-	$A^1\Pi_u$+	$B^1\Delta_g$
0.9952	-75.774(2)	-75.6576(2)	-75.6569(2)	-75.5927(4)
1.1196	-75.8944(6)	-75.8181(1)	-75.818(1)	-75.7636(2)
1.1818	-75.9153(1)	-75.8556(1)	-75.8552(4)	-75.8129(2)
1.2440	-75.9213(1)	-75.8755(4)	-75.8747(2)	-75.8409(2)
1.3062	-75.9165(3)	-75.8814(1)	-75.8812(2)	-75.860(2)
1.3684	-75.9037(1)	-75.8791(3)	-75.886(6)	-75.8634(2)
1.4928	-75.8694(1)	-75.8587(1)	-75.8573(2)	-75.8546(2)
1.6172	-75.8355(2)	-75.8278(2)	-75.8307(2)	-75.8353(2)
1.7416	-75.8090(7)	-75.7953(2)	-75.8089(2)	-75.817(3)
1.8660	-75.7862(3)	-75.7674(2)	-75.7869(2)	-75.7867(3)

Table S4: Total energies in atomic units predicted by the improved penalty-based VMC method using the Psiformer ansatz for triplet and quintet excited states along the carbon dimer dissociation curve. Bond lengths are given in Ångström

Bond length	$a^3\Pi_u-$	$a^3\Pi_u+$	$c^3\Sigma_u^+$	$b^3\Sigma_g^-$	$C^5\Pi_g$
0.9952	-75.6979(2)	-75.6989(8)	-75.7578(2)	-75.6514(2)	—
1.1196	-75.8573(8)	-75.8569(3)	-75.8646(1)	-75.7986(1)	—
1.1818	-75.8941(2)	-75.8938(1)	-75.8782(1)	-75.8458(1)	—
1.2440	-75.9132(7)	-75.9128(3)	-75.8772(1)	-75.8733(1)	—
1.3062	-75.9185(2)	-75.9178(1)	-75.8650(1)	-75.888(2)	—
1.3684	-75.9155(2)	-75.9152(2)	-75.8470(3)	-75.8913(2)	-75.7627(2)
1.4928	-75.8933(2)	-75.8933(2)	-75.8016(1)	-75.8807(1)	-75.7821(1)
1.6172	-75.8620(1)	-75.8622(7)	-75.7642(1)	-75.8583(1)	-75.7836(3)
1.7416	-75.8291(8)	-75.8291(8)	-75.7435(2)	-75.8314(2)	-75.7752(3)
1.8660	-75.7972(4)	-75.7972(1)	-75.729(1)	-75.8044(3)	-75.7631(7)

S1.4 Ethylene

Table S5: Total energies in atomic units predicted by the improved penalty-based VMC method using the Psiformer ansatz for the two lowest singlet states of the Ethylene pyramidalization.

Torsion angle	A_g^1	B_1^1u	Pyramidalization angle	A_g^1	B_1^1u
0	-78.5842(1)	-78.3129(1)	0	-78.4719(2)	-78.3826(8)
15	-78.5801(3)	-78.3143(2)	20	-78.4716(12)	-78.3853(1)
30	-78.5672(2)	-78.3298(1)	40	-78.4655(2)	-78.3925(4)
45	-78.5466(2)	-78.3492(2)	60	-78.4514(8)	-78.3989(2)
60	-78.5188(2)	-78.3656(5)	80	-78.4243(3)	-78.3979(5)
70	-78.4991(9)	-78.3731(9)	90	-78.4066(15)	-78.3910(3)
80	-78.4788(3)	-78.3742(8)	95	-78.3890(3)	-78.3940(1)
85	-78.4722(5)	-78.3787(2)	97.5	-78.3868(2)	-78.3889(7)
90	-78.4691(2)	-78.3804(2)	100	-78.3844(1)	-78.3835(11)
-	-	-	102.5	-78.3815(2)	-78.3761(1)
-	-	-	105	-78.3788(6)	-78.3707(5)
-	-	-	110	-78.3723(4)	-78.3570(1)
-	-	-	120	-78.3547(2)	-78.3312(2)

S2 Training hyperparameters

Table S6: Training hyperparameters used in this work.

	Pretrain steps	Pretrain target basis	Train steps	$\tilde{\alpha}$	β	Evaluation steps
Li, Be	1k	aug-cc-pVTZ	100k	2	1	5k
other atoms	1k	aug-cc-pVTZ	100k	4	1	5k
CH_2O	5k	aug-cc-pVTZ	100k	4	1	5k
C_6H_6	100k	cc-pVDZ	200k	1	1	5k
other molecules	1k	aug-cc-pVTZ	100k	4	1	5k
carbon dimer	1k	aug-cc-pVTZ	50k	4	1	5k
ethylene	1k	aug-cc-pVTZ	100k	4	1	5k

S3 Spin assigned wave functions in quantum Monte Carlo

Electronic wave functions $\Phi(\mathbf{x}_0, ..., \mathbf{x}_N)$ are functions of spacial and spin variables $\mathbf{x}_i = (\mathbf{r}_i, \sigma_i)$. Being fermions, electrons obey anti-symmetric statistics and electronic wave functions change sign upon the permutation of any two electrons:

$$\Phi(..., \mathbf{x}_i, ..., \mathbf{x}_j, ...) = -\Phi(..., \mathbf{x}_j, ..., \mathbf{x}_i, ...)\,. \tag{1}$$

If the studied Hamiltonian does not depend on spin explicitly, as is the case for the molecular Hamiltonian, it shares a common set of eigenstates with the total spin operator $\hat{S}^2$. The Hamiltonian and the total spin operator (as well as one of its components) can then be diagonalized independently, giving rise to a set of spacial and spin eigenfunctions. The spin eigenfunctions correspond to eigenvalues S (spin quantum number) and m_S (magnetic quantum number) and are linear combinations of products of the single electron spin indicator functions α and β. Because of the independence of the spacial and spin parts, the spin eigenfunctions have to be symmetric (anti-symmetric) upon exchange of particles, with the spacial wave function being anti-symmetric (symmetric), respectively. Eigenfunctions with spin quantum number S have a multiplicity of $2S + 1$, that is there exist $2S + 1$ degenerate solutions (singlet, doublet, triplet, ...), each with a different m_S value. For example, for a two particle system there are three symmetric (triplet) and one anti-symmetric (singlet) spin eigenfunctions:

$$\begin{aligned}
\zeta_{1,1} &= \alpha(1)\alpha(2) \\
\zeta_{1,0} &= \frac{1}{\sqrt{2}}(\alpha(1)\beta(2) + \beta(1)\alpha(2)) \\
\zeta_{1,-1} &= \beta(1)\beta(2) \\
\zeta_{0,0} &= \frac{1}{\sqrt{2}}(\alpha(1)\beta(2) - \beta(1)\alpha(2))\,,
\end{aligned} \tag{2}$$

where the two subscripts stand for the spin and magnetic quantum numbers, respectively.

When addressing the spin-independent molecular Hamiltonian, within VMC, it's often advantageous to optimize the spin-assigned spacial wave function rather than the fully anti-symmetric wave function. This approach imposes an anti-symmetry constraint for like-spin electrons (the spin component is generally symmetric under the exchange of same-spin particles), while eliminating the necessity to enforce symmetry requirements on opposite-spin particles. The convention is to assign spin-up to the first $N_\uparrow$ electrons and spin-down to the remaining $N - N_\uparrow = N_\downarrow$ electrons. While this effectively gives rise to distinguishable particles, spin independent expectation values are not affected by the assignment and full symmetry can be restored by multiplying with the spin function and anti-symmetrizing. It is worth noting that in the process of fixing the spin of the electrons the magnetic quantum number is also determined, as the difference between the number of spin up and spin down electrons. The spin quantum number on the other hand depends on the symmetry of the spacial and spin wave functions and is determined in the optimization process. During variational optimization, the spin assigned wave function will hence converge to the ground state among the wave functions compatible with the fixed m_S component. For the two electron example, picking a wave function with an equal number of spin-up and spin-down electrons allows expressing wave functions with all possible values of S ($\zeta_{1,0}$ and $\zeta_{0,0}$), while a wave function with two same-spin electrons restricts to the $S = 1$ spin sector (either $\zeta_{1,1}$ or $\zeta_{1,-1}$).
